# A signature of dynamic biogeography: enclaves indicate past species replacement

**DOI:** 10.1098/rspb.2017.2014

**Published:** 2017-11-29

**Authors:** B. Wielstra, T. Burke, R. K. Butlin, J. W. Arntzen

**Affiliations:** 1 Department of Animal and Plant Sciences, University of Sheffield, Sheffield S10 2TN, UK; 2 Department of Ecology and Evolutionary Biology, University of California, Los Angeles, CA 90095, USA; 3 Naturalis Biodiversity Center, PO Box 9517, Leiden, 2300 RA, The Netherlands; 4 Department of Marine Sciences, University of Gothenburg, Gothenburg 405 30, Sweden

**Keywords:** historical biogeography, hybrid zone movement, introgression, secondary contact, phylogeography, *Triturus*

## Abstract

Understanding how species have replaced each other in the past is important to predicting future species turnover. While past species replacement is difficult to detect after the fact, the process may be inferred from present-day distribution patterns. Species with abutting ranges sometimes show a characteristic distribution pattern, where a section of one species range is enveloped by that of the other. Such an enclave could indicate past species replacement: when a species is partly supplanted by a competitor, but a population endures locally while the invading species moves around and past it, an enclave forms. If the two species hybridize and backcross, the receding species is predicted to leave genetic traces within the expanding one under a scenario of species replacement. By screening dozens of genes in hybridizing crested newts, we uncover genetic remnants of the ancestral species, now inhabiting an enclave, in the range of the surrounding invading species. This independent genetic evidence supports the past distribution dynamics we predicted from the enclave. We suggest that enclaves provide a valuable tool in understanding historical species replacement, which is important because a major conservation concern arising from anthropogenic climate change is increased species replacement in the future.

## 1. Background

**Figure 1.**
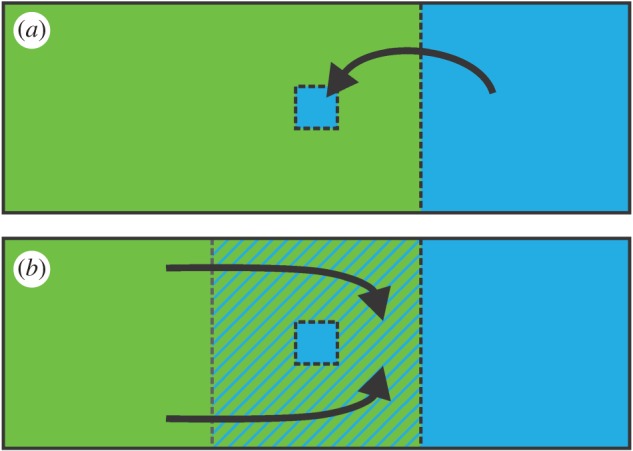
Two hypotheses on enclave formation in a pair of parapatric species. In (*a*) a blue species founds a population within the range of a green one via long-distance colonization, by leapfrogging across a stable contact zone. In (*b*) a relict population of a receding blue species persists locally within the range of a superseding green species, behind a moving contact zone, which was initially positioned at the grey dotted line. If the two species hybridize, a genomic footprint of hybrid zone movement would be expected under the moving contact zone scenario (in the hatched area in (*b*)), but not under the stable contact zone scenario. (Online version in colour.)

Species that meet in nature but geographically exclude one another have parapatric distributions [1]. While the position of parapatric range boundaries may be dynamic, their actual movement is a protracted process and has necessarily been recorded over shallow time frames only [1,2]. We here argue that the current spatial arrangement of parapatric species can be informative about distributional shifts in the more distant past. In particular, we highlight the insight provided by enclaves. An enclave is formed when part of the distribution of one member of a pair of parapatric species is isolated inside the range of the other (figure 1). An enclave could originate via colonization—analogous to a species establishing a peripatric population on an oceanic island [3,4]. Alternatively, the enclave might be a relict distribution patch of a previously broader distribution, left after incomplete species replacement—akin to rising sea levels disconnecting a continental island population from the mainland [5,6]. The likelihood of either scenario depends on the interplay between the mean and shape of a species’s dispersal kernel [7] and the enclave's distance from the main distribution range [8]. We assert that enclaves of species with low vagility, relative to the distance between the enclave and the main range, provide strong *a priori* support for species replacement. We provide proof of concept for an enclave system involving newts.

We take advantage of the fact that contact zones for parapatric species tend to correspond to hybrid zones [1]. When, upon secondary contact, a hybrid zone is first established, and one of the species possesses a competitive edge over the other one, this would result in a non-equilibrium phase of species replacement, with the hybrid zone moving as a consequence [1,4,5]. Furthermore, for a hybrid zone initially stabilized at an environmental gradient, this equilibrium phase could be disrupted if climate change shifts the balance in favour of one of the species [9]. If the species involved show introgressive hybridization, a moving hybrid zone is predicted to leave a trail of unlinked, selectively neutral alleles in its wake, derived from the displaced species and inside the territory newly claimed by the expanding one (initially based on theoretical principles [10], later supported by simulation [11] and recently backed up by empirical findings [12]). Under this rationale, the hypothesis of enclave formation can be tested independently in hybridizing species, based on the geography of genome-wide interspecific gene flow: an enclave formed by incomplete species replacement is expected to be accompanied by a genomic footprint of hybrid zone movement, while an enclave formed by colonisation is not consistent with this pattern (figure 1).

**Figure 2.**
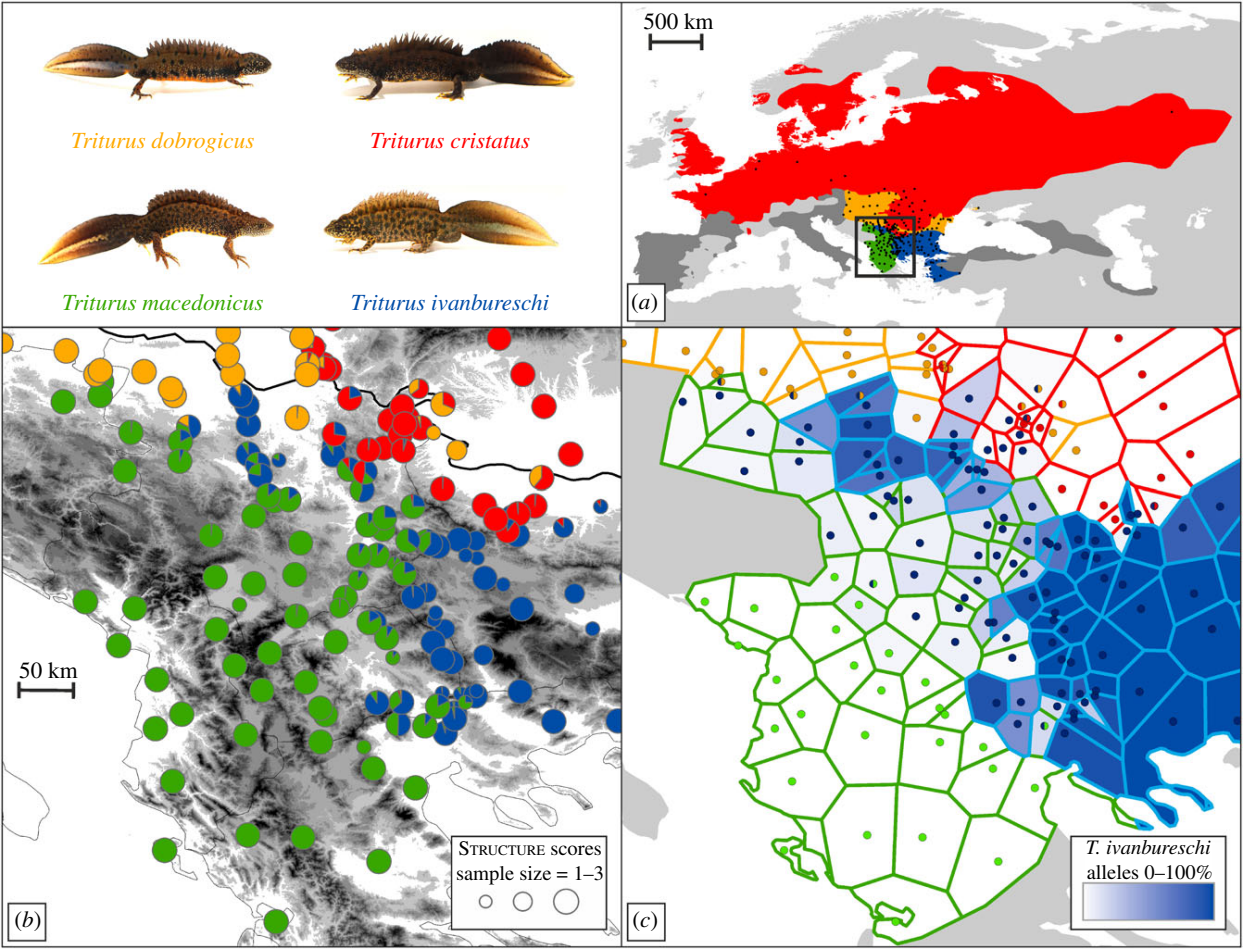
A *Triturus* enclave in south-eastern Europe. In (*a*) the range of the genus *Triturus* is shown, with approximate outlines of the ranges of the four species under study shown in colour (based on ref. [14]). The ranges of additional *Triturus* species are in dark grey. Dots are sampled localities. The box delineates part of the Balkan Peninsula, highlighted in (*b,c*). In (*b*) pie diagrams illustrate the average fraction of ancestry derived from the four species per locality, based on a Structure analyses of 52 nuclear DNA markers, with pie slices coloured according to species. Pie diameter indicates sample size. Grey shading reflects elevation. The thick black line denotes the Danube River, which connects the western and eastern segments of the range of *T. dobrogicus*. In (*c*) the colour of the border of Thiessen polygons specifies which species is the genetically dominant one, based on the Structure results. The blue shading of polygons reflects the proportion of alleles diagnostic for *T. ivanbureschi* present at a location (i.e. the average hybrid index). Dots reflect localities and are coloured according to mtDNA type. (Online version in colour.)

We examine an enclave observed in crested newts (Amphibia: *Triturus*), located in central Serbia, on the Balkan Peninsula [13]. Part of the range of *T. ivanbureschi* (blue in figure 2*a*) is detached from the main distribution, because the range of another species, *T. macedonicus* (green), intervenes. The ranges of two additional species, *T. cristatus* (red) and *T. dobrogicus* (orange), border that of *T. ivanbureschi* in the north. Both *T. ivanbureschi* and *T. macedonicus* colonized the central Balkan Peninsula postglacially, as they expanded from discrete glacial refugia, distant from the enclave [15]. *Triturus cristatus* was already in place as it had a glacial refugium in the southern protrusion of its current range, where it borders *T. ivanbureschi* today [16]. Although *T. dobrogicus* expanded its range postglacially [17], it is, uniquely among crested newts, a specialized lowland species [18], and its distribution in the south is bounded by an elevational ecotone (figure 2*b*). Therefore, while *T. cristatus* or *T. dobrogicus* represent a biological barrier north of the enclave, it is unlikely that either species invaded the range of *T. ivanbureschi* over a substantial area.

As the distance between the enclave and the main range of *T. ivanbureschi* (approx. 83 km) is over 20 times larger than lifetime dispersal distance (approx. 3.7 km [12]), incomplete species replacement (rather than long-distance colonization) is the likely explanation for the enclave's origin. Because crested newt species ranges meet at hybrid zones that facilitate introgression [19], we are in a position to test for a genomic footprint of hybrid zone movement around the enclave. We predict that such a genomic footprint was left by *T. ivanbureschi*, where we assume it was superseded by *T. macedonicus*. We do not predict a pronounced asymmetry in introgression between *T. ivanbureschi* and either *T. cristatus* or *T. dobrogicus*, as we expect *T. ivanbureschi*'s hybrid zones with these species to have been relatively stable.

## 2. Material and methods

### (a) Sampling

We included 1–3 individuals (mean 2.645) from 251 *Triturus* localities; 664 individuals in total (figure 2*a*; electronic supplementary material, table S1). For part of these individuals the full set of sequence data (*n* = 308) or (mt) DNAs (*n* = 283) were available from previous studies [12,17,20–24], while additional individuals (*n* = 73) from Serbia and Bulgaria are studied here for the first time. We selected 15 reference individuals per species—three individuals from five localities positioned away from contact zones and presumed to be unaffected by interspecific gene flow—to determine whether individual markers are diagnostic (electronic supplementary material, table S1). We created a Voronoi diagram in ArcGIS 10 (www.esri.com), where each locality is represented by a Thiessen polygon that contains the area that is closer to that particular locality than to any other one (figure 2*c*).

### (b) mtDNA sequencing and analysis

We Sanger sequenced a 658 bp mtDNA fragment for 625 individuals (following [17]) and were able to sequence a 110 bp internal fragment for 23 individuals following [12]. The clear-cut geographical distribution of mtDNA allowed us to infer mtDNA type for the 16 remaining individuals (electronic supplementary material, table S1). The 658 bp mtDNA fragments were added to the mtDNA haplotype database of [17] and collapsed into haplotypes with MacClade 4.08 [25]. To assign new haplotypes to species, we constructed a neighbour-joining phylogeny with 1000 bootstrap replicates in MEGA 5 [26], with the Pyrenean newt *Calotriton asper* and the marbled newt *T. marmoratus* (from [27]) as outgroups (electronic supplementary material, figure S1; table S2). Internal 110 bp mtDNA fragments were aligned with the set of 658 bp haplotypes and this set was then trimmed accordingly. By removing redundancy in MacClade, the internal fragments could be allocated unambiguously to species-diagnostic mtDNA type.

### (c) Sequencing nuclear DNA

For all 664 individuals, we sequenced 52 nuclear markers following [22]. Details are in electronic supplementary material, text S1.

### (d) Bayesian clustering analysis

We used Structure 2.3.3 [28] to estimate the fraction of ancestry for each individual derived from the four parental species based on nuclear DNA data. We used the admixture model in combination with the correlated allele frequency model with 1 000 000 iterations, after 250 000 iterations of burn-in, and ran 10 replicates. An initial Structure analysis on the set of reference individuals, in which we allowed the number of genepools *k* to vary from 1–20 (with the upper limit defined by the total number of localities included), confirmed *k* = 4 as the most likely number of genepools under Evanno's Δ*k* criterion [29], as implemented in Clumpak [30]. Next we conducted a Structure run on the full set of individuals, in which we fixed *k* to 4. The lowest *Q*-value with which a reference individual was allocated to its respective species was *Q* = 0.9794. Localities allocated to two (or in some cases three) species with *Q* > 0.0206 (i.e. 1–0.9794) were considered genetically admixed. Structure scores (with replicates summarized in Clumpak) are presented in electronic supplementary material, table S1 and locality averages are plotted in figure 2*b*.

### (e) Hybrid index, heterozygosity and ancestry, and test for asymmetric introgression

**Figure 3.**
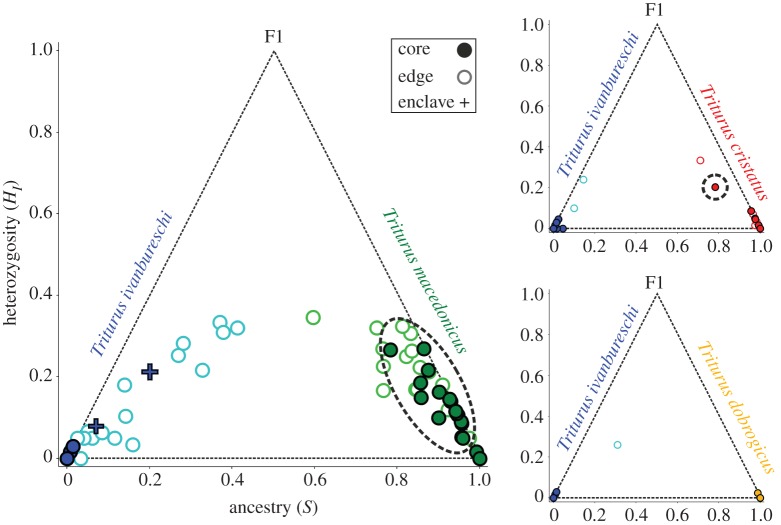
Heterozygosity versus ancestry plots, comparing *Triturus ivanbureschi* with three congeners. The left and right corners of the triangle correspond to the two parental species and the upper corner to an F1 hybrid. Circles represent localities and are coloured according to the genetically dominant species based on the Structure analysis. We distinguish between localities from the core of the range (dark, filled circles), i.e. localities of which the Thiessen polygon (figure 2*c*) only borders others in which the same species is genetically dominant (based on Structure results), or from the edge of the range (light, open circles), i.e. localities for which the Thiessen polygon borders at least one locality where the alternative species is genetically dominant. Many *T. macedonicus* core localities show introgression of *T. ivanbureschi* alleles (highlighted with dashed ellipse) but core localities of other species (except a single *T. cristatus* one) do not show such introgression. The blue crosses represent *T. ivanbureschi* core localities from the enclave. (Online version in colour.)

We determined the evolutionary origin of alleles based on our 15 reference individuals per species (see section on sampling). We found 23 nuclear DNA markers to be diagnostic, i.e. exhibiting fixed allelic differences between *T. ivanbureschi* (the species with the enclave) and the other three species. We determined the proportion of diagnostic *T. ivanbureschi* alleles present at each locality, i.e. the mean hybrid index (electronic supplementary material, table S1), and plotted this on a map (figure 2*c*). For pairwise species comparisons of *T. ivanbureschi* versus the other three *Triturus* species, we determined individual heterozygosity (the fraction of markers heterozygous for alleles from each parental species) and ancestry (the fraction of alleles derived from each parental species) using the R [31] package ‘HIest’ [32]. Locality averages (electronic supplementary material, table S3) were plotted in Statistica 7 (www.statsoft.com) (figure 3). To test if introgression between species pairs was significantly asymmetrical we determined, for each locality, whether there was introgression of nuclear DNA alleles and what the fraction of introgressed alleles was (electronic supplementary material, table S3). We used a Fisher's exact test for the presence/absence data and a one-tailed Mann–Whitney *U* test for the quantitative data (electronic supplementary material, table S5). To avoid the confounding effects of gene flow from other *Triturus* species, we excluded localities showing ancestry of additional *Triturus* species (based on the Structure analysis or, in the case of low frequency introgression, inferred from the genetic composition of neighbouring Thiessen polygons; electronic supplementary material, table S4) from pairwise comparisons.

### (f) Geographical cline analysis

**Figure 4.**
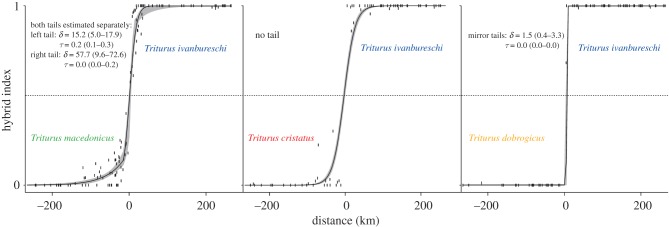
Geographical cline analysis, comparing *Triturus ivanbureschi* with three congeners. Two-dimensional sampling was collapsed into a one-dimensional transect, based on the shortest distance of localities to a 0.5 hybrid index contour. Geographical clines were fitted to the mean hybrid index for each locality. The 95% credible cline region is shown in grey and the sampling sites are denoted by vertical bars. The dotted line signifies a hybrid index of 0.5. The chosen cline models, with tail parameters if relevant, are noted for each cline fit. The tail parameters *δ* and *τ* represent the distance from the cline centre to the tail and the tail slope. Two-log-likelihood-unit support limits are presented in parentheses. (Online version in colour.)

In pairwise species comparisons for *T. ivanbureschi* versus the other three species, excluding localities showing ancestry of additional *Triturus* species (electronic supplementary material, table S4), we determined the 0.5 contour for the hybrid index using the ‘akima’ package [33] in R. We calculated the minimum straight-line distance of each locality from that contour in ArcGIS, and gave distances for *T. ivanbureschi* a positive sign and distances for other species a negative sign (electronic supplementary material, table S3). For the resulting one-dimensional transect [34] we then fitted a geographical cline to the hybrid index in the R package ‘HZAR’ [35]. Frequency was fixed at the ends of the cline to 1 (pure *T. ivanbureschi*) and 0 (pure for the other species). The hybrid index is the proportion of diagnostic alleles, calculated from 23 diploid markers (minus a few missing genotypes) in one to three individuals. Therefore, it was fitted with binomial error distribution and sample size equal to the total number of genotypes (loci × individuals) per locality. Although markers are unlikely to be closely linked, they may not behave independently. Using the number of genotypes rather than the number of alleles makes our test conservative, despite this constraint. We tested five models, differing in complexity: no tail, left tail only, right tail only, mirror tails or both tails estimated separately. We used the lowest Akaike information criterion score corrected for small sample size to select the best one; in each case the score of the chosen model was at least two likelihood points lower than that of the next best fitting model (figure 4; electronic supplementary material, table S6).

## 3. Results

Bayesian clustering analysis with Structure based on 52 nuclear DNA markers allows us to delineate the enclave of *T. ivanbureschi* (figure 2*b*). The range of *T. macedonicus* penetrates north-eastwards, into the range of *T. ivanbureschi* and right up to the range of *T. cristatus*, isolating the *T. ivanbureschi* enclave. Several localities between the enclave and the main range of *T. ivanbureschi* show genetic admixture among all three species. A closer look at the 23 nuclear DNA markers that are diagnostic for *T. ivanbureschi* versus the three other species reveals a substantial number of *T. ivanbureschi* alleles in the core of the range of *T. macedonicus*, but not the other way around (figure 2*c*). Similarly, the mtDNA of *T. ivanbureschi* protrudes into the range of *T. macedonicus* (figure 2*c*). No such asymmetry is apparent when comparing *T. ivanbureschi* with *T. dobrogicus*, while foreign alleles reach inside the range of *T. cristatus* only over a short distance, just north of the enclave. Plots of heterozygosity versus ancestry support the pattern of *T. ivanbureschi* alleles reaching further into the range of *T. macedonicus* than the ranges of the other two species (figure 3).

The asymmetry in nuclear DNA introgression from *T. ivanbureschi* into *T. macedonicus* is statistically significant, based on either the presence (Fisher's exact test, *p* = 0.000) or the intensity (one-tailed Mann–Whitney *U* test, *U* = 1423.5; *Z* = −3.11; *p* = 0.000) of introgression. There is no such asymmetric nuclear DNA introgression into *T. cristatus* (*p* = 0.554; *U* = 1071.0; *Z* = −0.77; *p* = 0.341) or *T. dobrogicus* (*p* = 1.000; *U* = 810.5; *Z* = 0.47; *p* = 0.444). To remove the influence of a potential bias in sampling, we collapse the two-dimensional sampling into a one-dimensional transect, based on the shortest distance of localities to a 0.5 hybrid index contour. One-dimensional geographical clines, fitted to a hybrid index derived from the 23 diagnostic nuclear DNA markers for *T. ivanbureschi* versus each of the other three species, differ in the models that best fit the data. Only for *T. macedonicus* was an asymmetric cline model preferred, with a substantial introgression of *T. ivanbureschi* alleles into *T. macedonicus*, but not in the opposite direction (figure 4).

## 4. Discussion

Guided by the enclave observed in our crested newt system, we hypothesized a past parapatric range shift, in which *T. macedonicus* intersected the range of *T. ivanbureschi* and excised the enclave. As the two species must have hybridized in the process, we could test this scenario by exploiting the pattern of interspecific gene flow. In line with predictions for moving hybrid zones based on theory [10] and data simulation [11], we exposed a genomic footprint of hybrid zone movement, left by *T. ivanbureschi*, where it has been replaced by *T. macedonicus*. While cytonuclear incompatibilities or dispersal effects might cause asymmetry in mtDNA introgression and selection might cause asymmetry for any given locus (while not necessarily in the same direction), a genome-wide shared asymmetry in introgression over an extensive area strongly favours a scenario of species displacement with hybridization [12]. Hence, the crested newt case illustrates the predictive power of enclaves for inferring past species replacement.

Enclaves are not often reported, but, considering that three are known in *Triturus* newts alone (see [36,37] for two other species pairs), this does not necessarily mean that they are rare. Enclaves may well be transient, with species replacement not prevented but merely postponed locally. Indeed, all individuals from the *T. ivanbureschi* enclave show some degree of genetic admixture with other crested newt species. The ephemeral nature of enclaves is illustrated by a previously documented enclave (established from museum specimens) having been taken over by an invader over a period of roughly 50 years [38]. Furthermore, ‘islands of alleles’ derived from a displaced species that was genetically swamped out by a competitor, could be regarded as genetic fossils of a past enclave [39]. In general, being small and isolated from the main distribution makes enclave populations more susceptible to the extinction risks associated with habitat fragmentation [40].

Once an enclave is identified, it can provide crucial insights into historical biogeography. While hybrid zones have been tracked at the scale of decades [4], they are thought to stabilize quickly at environmental density troughs [10,41]. Enclaves, however, indicate hybrid zone movement over extended periods of time—an opportunity that has as yet received little-to-no attention in the hybrid zone literature [4,11]. Understanding past shifts in the mutual range boundaries of parapatric species is particularly relevant in light of the expected future increase in such shifts in response to man-made habitat alteration and climate change [9,42].

## Supplementary Material

Supplementary Text S1 and Figure S1

## Supplementary Material

Supplementary Tables S1-S6

## Data accessibility

The data underlying this paper are archived in the Dryad Digital Repository (https://doi.org/10.5061/dryad.9k1h0) [43].
