# Supplementary material for: A signature of dynamic biogeography: enclaves indicate past species replacement
Source: Proc Biol Sci. 2017 Nov 29;284(1868):20172014. doi: 10.1098/rspb.2017.2014 (PMC5740283; doi:10.1098/rspb.2017.2014)

Text S1: Extended methods on nuclear DNA sequencing.

For all 664 individuals, we sequenced 52 nuclear markers following the protocol presented by Wielstra *et al.* [1]. In brief, we amplified markers of c. 140 bp in length, designed from transcriptome data and positioned in the 3' untranslated regions of protein coding genes, in five multiplex PCRs. We pooled the multiplexes for each individual and ligated unique tags. Amplicons were sequenced on the Ion Torrent next-generation sequencing platform. The output was processed with a bioinformatics pipeline. First, poor quality reads were filtered out by removing those with a length less than 100 bp and an average quality of less than Q20. Next, reads for each individual were mapped against the targeted nuclear markers using BWA v0.7.3 [2] and SNP/InDel calling was performed with SAMtools v0.1.18 [3]. A SAMtools quality score over Q60 was required for SNP/InDels to be retained. Subsequently, alleles were reconstructed by determining the combination of SNPs and InDels in the reads of marker-individual combinations. Marker-individual combinations were required to have at least ten reads, alleles were required to be present in at least 25% of the reads, and a maximum of two alleles were allowed to be present, otherwise a marker-individual combination was considered failed. Finally data was converted into a genotypic data format by recoding the different allelic variants for each marker to a unique integer, resulting in two integers per individual per marker. Mean coverage was 851.0 (range 0–94,556) per marker-individual combination and 97.9% of marker-individual combinations were considered successful (meaning they had at least 20 reads available).

References:

[1] Wielstra, B., Duijm, E., Lagler, P., Lammers, Y., Meilink, W.R.M., Ziermann, J.M. & Arntzen, J.W. 2014 Parallel tagged amplicon sequencing of transcriptome-based genetic markers for *Triturus* newts with the Ion Torrent next-generation sequencing platform. *Mol. Ecol. Resour.* **14**, 1080-1089. (doi:10.1111/1755-0998.12242).
[2] Li, H. & Durbin, R. 2009 Fast and accurate short read alignment with Burrows–Wheeler transform. *Bioinformatics* **25**, 1754-1760. (doi:10.1093/bioinformatics/btp324).
[3] Li, H., Handsaker, B., Wysoker, A., Fennell, T., Ruan, J., Homer, N., Marth, G., Abecasis, G., Durbin, R. & Subgroup, G.P.D.P. 2009 The Sequence Alignment/Map format and SAMtools. *Bioinformatics* **25**, 2078-2079. (doi:10.1093/bioinformatics/btp352).

Fig. S1. Neighbour-joining phylogeny for *Triturus* mtDNA. The outgroup and bootstrap support values below 80% are not shown. Haplotype codes correspond to Table S2. The mtDNA haplotypes of the different *Triturus* species cluster in unambiguously supported and genetically highly distinct monophyletic groups. Hence, determining the species of origin of mtDNA haplotypes is straightforward.

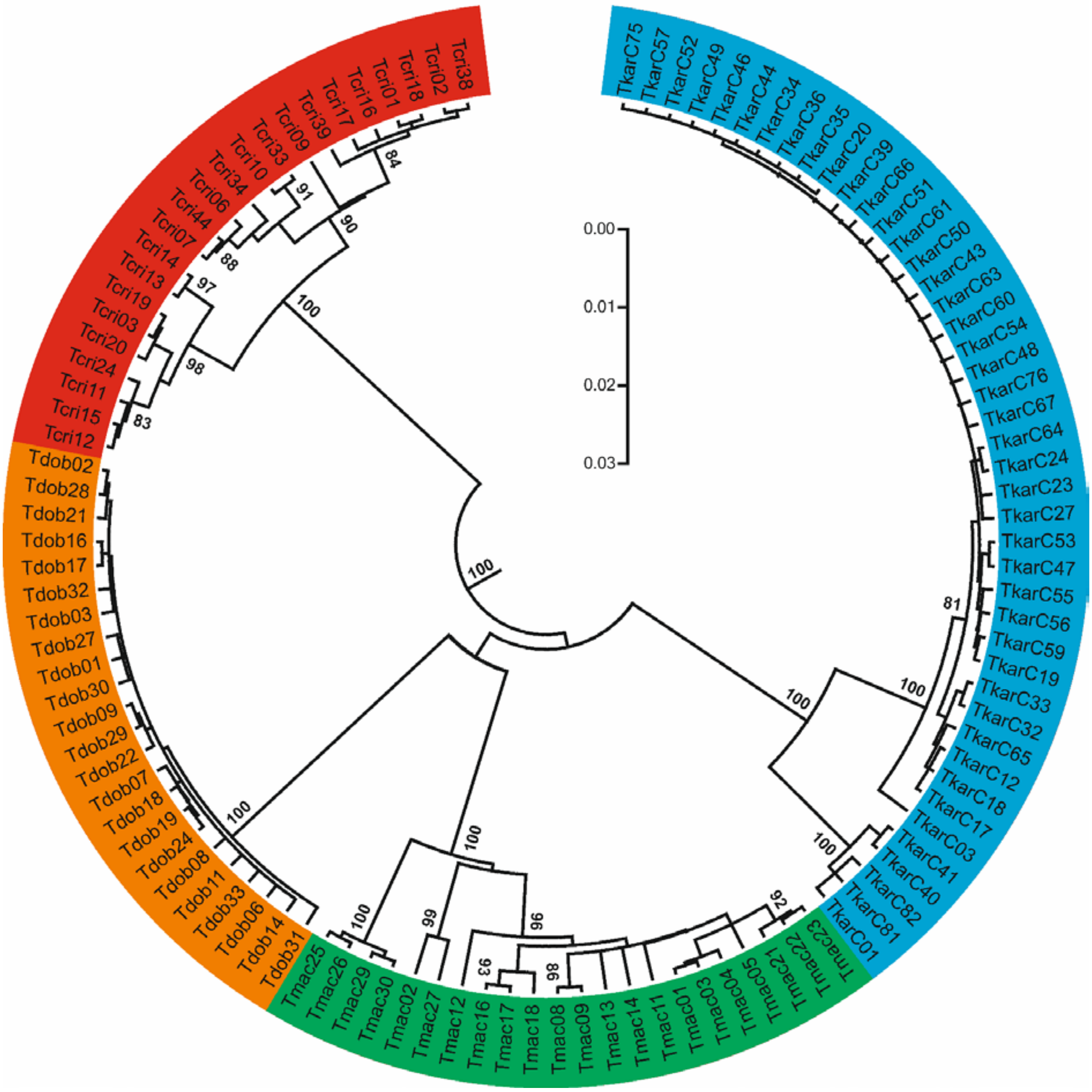

Supplement: Supplementary Text S1 and Figure S1 [file rspb20172014supp1.pdf]
